# Oral Exposure to House Dust Mite Activates Intestinal Innate Immunity

**DOI:** 10.3390/foods10030561

**Published:** 2021-03-09

**Authors:** Sara Benedé, Leticia Pérez-Rodríguez, Mónica Martínez-Blanco, Elena Molina, Rosina López-Fandiño

**Affiliations:** Instituto de Investigación en Ciencias de la Alimentación (CIAL, CSIC-UAM), 28049 Madrid, Spain; s.benede@csic.es (S.B.); leticia.p.r@csic.es (L.P.-R.); m.martinez.blanco@csic.es (M.M.-B.); e.molina@csic.es (E.M.)

**Keywords:** food allergy, house dust mite, intestinal epithelial cells, dendritic cells, group 2 innate immune cells

## Abstract

Scope: House dust mite (HDM) induces Th2 responses in lungs and skin, but its effects in the intestine are poorly known. We aimed to study the involvement of HDM in the initial events that would promote sensitization through the oral route and eventually lead to allergy development. Methods and results: BALB/c mice were exposed intragastrically to proteolytically active and inactive HDM, as such, or in combination with egg white (EW), and inflammatory and type 2 responses were evaluated. Oral administration of HDM, by virtue of its proteolytic activity, promoted the expression, in the small intestine, of genes encoding tight junction proteins, proinflammatory and Th2-biasing cytokines, and it caused expansion of group 2 innate immune cells, upregulation of Th2 cytokines, and dendritic cell migration and activation. In lymphoid tissues, its proteolytically inactivated counterpart also exerted an influence on the expression of surface DC molecules involved in interactions with T cells and in Th2 cell differentiation, which was confirmed in in vitro experiments. However, in our experimental setting we did not find evidence for the promotion of sensitization to coadministered EW. Conclusion: Orally administered HDM upregulates tissue damage factors and also acts as an activator of innate immune cells behaving similarly to potent oral Th2 adjuvants.

## 1. Introduction

Allergic diseases are commonly described as a failure to achieve the otherwise normal tolerance state. Even if the molecular mechanisms involved in sensitization are still poorly understood, evidence indicates that environmental factors play an important role in the sensing of innocuous antigens and the development of the Th2 immune responses that characterize the allergic condition [1]. In this respect, the fact that many common airborne allergens, such as house dust mite (HDM), fungi, and cockroach, contain proteolytic enzymes has prompted the hypothesis that proteolytic activity represents an important mechanism of initiation of allergic inflammation [2]. 

HDM is a ubiquitous aeroallergen that promotes a potent Th2-type airway inflammation in asthmatic subjects [3]. HDM is a complex mixture of constituents that comprises proteases belonging to the serine protease family, which include well-known allergens such as *Dermatophagoides pteronyssinus* (Der p) 3, 6, and 9; and to the cysteine protease family, such as Der p 1 [4]. Protease-induced breakdown of epithelial barriers through the cleavage of tight junctional proteins that maintain barrier function has been associated with exacerbated responses to allergens that trigger allergic diseases [2,5]. Besides, HDM proteases promote sensitization through the stimulation of protease-activated receptors (PARs) widely distributed on the cells of the airways, where they contribute to the inflammation characteristic of allergic diseases through the opening of tight junctions and the production of cytokines, chemokines, and growth factors [6]. Nevertheless, HDM can also induce expression of proinflammatory cytokines and chemokines in bronchial epithelial cells independently of its protease activity [7].

On the other hand, HDM particles contain pathogen-associated molecular patterns (PAMPs) that function as immune adjuvants acting on pattern recognition receptors (PRRs), which are important components of innate immunity required for the activation of antigen-presenting cells such as dendritic cells (DCs) [8]. Among the PRR ligands found in HDM, Der p 2 has structural and functional sequence homology with the LPS component which binds to TLR4, the PRR for endotoxins [9]. On the other hand, HDM chitin, a long chain polymer of N-acetylglucosamine, activates the innate immune system via TLR2 and C-type lectin, thereby inducing Th2 responses in lungs and epithelial cells from skin [10]. 

In addition to the airways, HDM is also present in the gastrointestinal tract of humans and mice, where it disrupts tight junction proteins and impairs the mucus layer at the colonic level [11]. As a consequence, HDM increases epithelial permeability and induces the production of proinflammatory and anti-inflammatory cytokines. These effects, linked to its proteolytic activity, were found independent of previous sensitization to HDM or of Th2 immunity [11]. However, oral exposure of mice to Der p through breast milk strongly induces susceptibility to allergic asthma [12] and several cases of systemic allergy due to the ingestion of mite contaminated food have been reported [13]. Therefore, the question arises as to whether HDM might promote gastrointestinal sensitization by enabling its delivery through the disruption of tight junctions, its interaction with intestinal epithelial cells (IECs), and/or the activation of DCs; or even act as an adjuvant towards sensitization to a bystander protein, eventually favoring allergy, not only to the proteins of the mite themselves, but also to food proteins [13]. 

The intestinal epithelium forms the first structural barrier against food allergens. The epithelial barrier function is mainly maintained by the formation of tight junctions, composed of zonula occludens 1–3, occludin and claudins 1–5 [14]. As a reaction to stress or cell injury, IECs release alarming cytokines such as IL-33, IL-25, and TSLP, which expand group 2 innate lymphoid cells (ILC2s) that produce IL-4, IL-5 and IL-13 [15]. These cytokines prime DCs to induce a Th2 phenotype on T cells and also directly stimulate adaptive Th2 immunity and subsequent allergic reaction [1]. In this work, we aimed to evaluate if orally administered HDM could affect the early events of sensitization to food by the assessment of markers of the integrity and function of the intestinal barrier, the proportion of lamina propria ILC2s, the specialization of DCs and the induction of effector T cell responses. For this purpose, we compared the effects in mice of proteolytically active and inactive HDM, as such, or in combination with a common food allergen such as egg white (EW).

## 2. Experimental Section

A house dust mite extract, low in endotoxin and with a known content of Der p 1 (10.2 μg.mg^−1^ dry weight), was purchased from Citeq Biologics (Groningen, The Netherlands). EW was carefully separated from fresh eggs and lyophilized. Its protein content, lipid composition, and absence of cross-contamination were examined as in Pablos-Tanarro et al. [16]. The samples were dissolved in sterile phosphate-buffered saline (PBS; pH 7.4) and the absence of lipopolysaccharide (LPS) was confirmed by using the transfected cell line THP1-XBlue™, stably expressing an NF-kB/AP-1-inducible secreted alkaline phosphatase reporter (SEAP) assessed by QUANTI-Blue™ assay (InvitroGen, Carlsbad, CA, USA), following the manufacturer’s instructions.

### 2.1. Inactivation of The House Dust Mite Extract

The proteolytically inactive extract (iHDM) was obtained by treatment with the serine protease inhibitor 4-(2-aminoethyl) benzenesulfonyl fluoride hydrochloride (AEBSF) and the cysteine protease inhibitor E-64 (both from Sigma-Aldrich, San Luis, MO, USA), at concentrations of 100 and 10 mM, respectively, at 37 °C for 30 min. Excess of inhibitors was removed using PD-10 desalting columns (GE Healthcare, Chicago, IL, USA). Its proteolitically active counterpart (HDM) was mock-treated by using a similar desalting procedure, although it was not previously incubated with protease inhibitors [11]. Protein concentration was determined using the bicinchoninic acid assay (Thermo Fisher Scientific, Waltham, MA, USA), following the manufacturer’s instructions. 

### 2.2. Protease Activity of the House Dust Mite Extract

The protease activity of HDM and iHDM was assessed using the fluorogenic peptide substrate Boc-Gln-Ala-Arg-AMC (Boc: N-tert-butoxycarbonyl; AMC: 7-amino-4-methylcoumarin) as previously described [17]. Briefly, a known amount of extract (with a final concentration of Der p 1 of 10 nM) was added to the substrate (100 µM) in 100 mM sodium phosphate buffer (pH 6.0) containing 10 mM EDTA and 1 mM dithiothreitol (DTT). Fluorescence produced by the hydrolysis of the substrate was monitored at 37 °C using a Fluorostar Optima microplate-reader (BMG LabTech, Ortenberg, Alemania) with excitation at 380 nm and emission at 460 nm. 

To assess the influence of protease inhibitors, present in EW in the proteolytic activity of the extract, protease activity was measured in mixtures of HDM and EW in different proportions, including the same proportion as given to the mice (100 µg of HDM:50 mg of protein in 200 µL). Pea extract (The HealthyTree Company, Bicester, UK), whey protein (Arla Foods Ingredients, Sønderhøj, Denmark), and protease-free bovine serum albumin (Sigma-Aldrich) were similarly used to test if the effect of EW on the proteolytic activity of HDM was specific or due to the presence of protease inhibitors. The effect of pH on the activity of HDM was also evaluated following incubation of the extract at 37 °C for 1 h in 100 mM sodium phosphate buffer at pH 2.0, 3.5, and 7.0 before protease activity was measured.

### 2.3. Hydrolysis of Ovalbumin by House Dust Mite

HDM was incubated with ovalbumin in the same proportion as given to the mice (100 µg of HDM:50 mg of protein in 200 µL). After 24 h at 37 °C the serine and cysteine protease inhibitors, AEBSF and E-64, were added and samples were analyzed by reversed phase high performance liquid chromatography according to Benedé et al. [18]. In this experiment, ovalbumin (which amounts to, approximately, 54% of the protein content in EW) was preferred over EW because it allowed the detection of a low degree of protein degradation.

### 2.4. Experiments in Mice

Female 6 week-old BALB/c mice (Charles River Laboratories, Wilmington, MA, USA), distributed in six groups of six mice each, received by intragastric gavage for six consecutive days: 200 µL of PBS, 100 µg of HDM, 100 µg of iHDM, 50 mg of EW, 50 mg of EW plus 100 µg of HDM; or 50 mg of EW plus 100 µg of iHDM (all dissolved in 200 µL of PBS). Although there are no data on the amount of HDM present in foods, which is likely to vary widely depending on environmental and storage conditions, the dose of HDM administrated to mice followed other authors who used between 20 and 100 μg [11,19]. On the sixth day, mice were given a second dose 2 h after the first one and sacrificed by CO_2_ inhalation 2 h later. Duodenum, jejunum, Peyer’s patches (PPs) and mesenteric lymph nodes (MLNs) were individually collected, snap frozen in liquid nitrogen and stored at −80 °C for gene expression analyses. For flow cytometry analyses of ILC2s, DCs and Th2 cells, the entire small intestine was collected for lamina propria cell isolation and MLNs were crushed through a 70 μm cell strainer (BD labware, Franklin Lakes, NJ, USA) and washed to obtain a single cell suspension. All protocols followed the European legislation (Directive 2010/63/EU) and were approved by Comunidad de Madrid (Ref PROEX 089/15).

### 2.5. Isolation of Lamina Propria Cells

The small intestine, after the removal of PPs and MLNs, was washed with Hanks’ Balanced Salt solution (HBSS, Corning Inc., New York, NY, USA) containing 2% of heat-inactivated fetal bovine serum (FBS HI) and cut into small pieces. After incubation of the pieces with HBSS containing 5% of FBS HI and 1 mM DTT for 20 min at 37 °C, they were transferred to a new tube and incubated with 1.3 nM EDTA in PBS for 10 min at 37 °C. Then, tissue pieces were washed three times with RPMI and digested with 1 mg/mL of collagenase D and 5 mg/mL of DNase (both from Roche, Basel, Switzerland) in RPMI, with 5% of FBS HI for 40 min at 37 °C. Once washed with RPMI and centrifuged at 200× *g* for 8 min, the pellet was suspended in 44% percoll and underlaid with 66% percoll. Interface cells were collected after centrifugation at 1000× *g* for 20 min and washed with RPMI. 

### 2.6. Bone Marrow Derived Dendritic Cells

DCs were differentiated from bone marrow cells as described previously [20]. Briefly, bone marrow cells were isolated from femurs of naïve BALB/c mice and cultured for 10 days in RPMI 1640 medium supplemented with FBS, L-glutamine, and penicillin/streptomycin (all from Biowest SAS, Nuaillé, France), containing 20 ng/mL GM-CSF (Peprotech, London, UK). Different concentrations (1–100 µg/mL) of HDM or iHDM were incubated with 1 × 10^6^ bone marrow-derived DCs (BM-DCs) for 24 h. Cells were analyzed by flow cytometry and stored at −80 °C for gene expression analyses.

### 2.7. Gene Expression Analyses

RNA was extracted from duodenum, jejunum, PPs, and MLNs of mice and from bone marrow-derived DCs using the NucleoSpin RNA Kit (Macherey-Nagel Gmbh and Co., Düren, Germany), according to the manufacturer’s instructions. RNA concentration and integrity were assessed in a NanoDrop 1000 (Thermo Fisher Scientific). First-stranded cDNA was synthetized using the PrimeScript RT reagent kit (TaKaRa Bio Inc., Shiga, Japan). RT-PCR was performed on a real-time thermocycler (ViiA7 Real-Time PCR System; Applied Biosystems, Foster, CA, USA), with SYBR Premix ExTaq II (TaKaRa). The specific primers are described in Supplementary Table S1. Data were normalized to the expression of the Actb gene (coding for *β*-actin) and relative quantification was performed using the comparative threshold cycle method (2-∆∆Ct). All amplifications were carried out in triplicate.

### 2.8. Flow Cytometry Analyses 

Cells stained with LIVE/DEAD^®^ Fixable Near-IR Dead Cell Stain Kit (Thermo Fisher Scientific) were blocked with anti-CD16/32 antibody (eBioscience, Waltham, MA, USA) and stained with specified antibodies. Antibodies used were those recognizing CD19 (FITC, clone MB19-1), hematopoietic lineage (FITC, (CD3, clone 17A2; CD45R, clone RA3-6B2; CD11b, clone MI/70; TER-119, clone TER119; Ly-G6, clone RB6-BC5; CD19, clone eBio1D3)), CD45.2 (PE-Cy7, clone 104), KLRG (APC eFluor 780, clone 2F1), ICOS (APC, clone C398.4A), ST2 (PE, clone RMST2-2), CD4 (Alexa Fluor 700, clone GK1.5), CD69 (PerCP-Cy5.5, clone H1.2F3), CD11c (PE-Cy7, clone N418), CD11b (Alexa Fluor 700, clone M1/70), CD64 (APC, clone X54-5/7.1), CD103 (PE, clone 2E7), MHCII (FITC, clone M5/114.15.2), CD86 (APC and PE, clone GL1) (all from eBioscience). Cells were acquired with a Gallios flow cytometer (Beckman Coulter, Brea, CA, USA) with two solid state lasers of standard colors red and blue and an eight-color system. Data were analyzed using FlowJo software v. 7.6.5, (TreeStar Inc., San Carlos, CA, USA).

### 2.9. Statistical Analyses

Statistical analyses were performed using the GraphPad Prism software, version 6.0 (Graph-Pad Software Inc., San Diego, CA, USA). A Mann–Whitney U test was used for determining statistical significance of gene expression data tissues and two-tailed unpaired Student’s *t*-test was used to assess differences of flow cytometry data from mice. Two-tailed paired Student’s *t*-test was used to assess differences of bone marrow derived DCs (BM-DCs). Results were expressed as the mean ± SEM.

## 3. Results

### 3.1. Oral Exposure to Proteolytically Active House Dust Mite Changes the Expression of Genes Related to Intestinal Epithelial Barrier Integrity 

**Figure 1:**
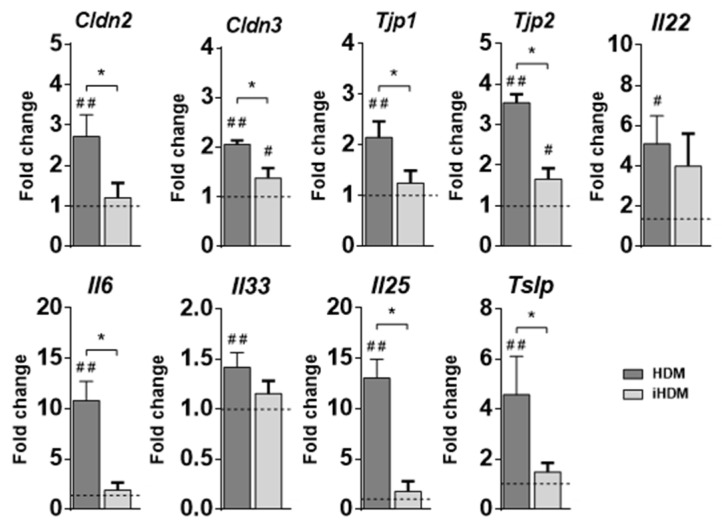
Relative gene expression of *Cldn2*, *Cldn3*, *Tjp1*, *Tjp2*, *Il22*, *Il6*, *Il33*, *Il25*, and *Tslp* determined in the jejunum of mice administered intragastrically proteolytically active or inactive house dust mite (respectively, HDM and iHDM) for six consecutive days. Gene expression was normalized to the reference gene *Actb* and the mouse group administered PBS, used as calibrator, is represented as a discontinuous line in the figure. Data are expressed as means ± SEM (*n* = 6). Pounds and asterisks indicate, respectively, statistically significant differences with respect to mice administered PBS or between both experimental groups. * and # *p* < 0.05, ## *p* < 0.01.

Initially, we evaluated the effect of the administration of HDM and iHDM on the expression of genes that code for factors that control intestinal permeability and mucosal protection, such as the tight junction molecules claudin-2, claudin-3, zonula occludens-1 and zonula occludens-2 (respectively, *Cldn2, Cldn3, Tjp1*, and *Tjp2*) [21]; Muc2 (*Muc2*), the main mucin of the mucus layer [22]; and IL-22 (*Il22*), a cytokine that promotes epithelial integrity, but also induces inflammatory effects under the influence of a proinflammatory environment [23]. The expression of *Cldn2*, *Cldn3*, *Tjp1*, *Tjp2*, and *Il22* was increased in the jejunum of mice fed HDM (Figure 1). Inactivation of the extract with serine and cysteine protease inhibitors either abolished or considerably reduced the effects of HDM, although the expression of *Il22* was not significantly lower in iHDM-treated mice (Figure 1). Expression of *Muc2* was not affected in either case (not shown). A similar response, except for the observation that *Tjp2* was unchanged, was observed in the duodenum (Figure S1). In addition, the expression of *Il6*, coding for the proinflammatory mediator IL-6, was also enhanced in the jejunum of mice fed HDM, but not in those receiving the inactivated extract (Figure 1).

IECs release cytokines, namely IL-33, IL-25, and TSLP (respectively encoded by *Il33*, *Il25*, and *Tslp*), that work as immune alarm signals in response to cellular stress or injury and are involved in the pathophysiology of allergic disease [15]. Administration of HDM upregulated the expression of *Il33, Il25*, and *Tslp* in the jejunum of mice (Figure 1). Expression of *Il33* and *Tslp* was also enhanced in the duodenum (Figure S1). This effect was associated with the proteolytic activity of HDM, since oral exposure of mice to iHDM did not modify *Il33*, *Il25*, or *Tslp* expression. 

The above-mentioned results suggest that HDM retains its proteolytic activity along its passage through the gastrointestinal tract. Given that gastric pH in mice is slightly higher than in humans [24,25] we tested the effect of pH 3.5 and 2.0 (characteristic of the fasted stomach of infants and adults, respectively) in vitro. The results confirmed that HDM proteases are able to resist for up to 30 min at pH 2.0 (Figure S2).

### 3.2. House Dust Mite Increases Intestinal Th2 Responses

**Figure 2:**
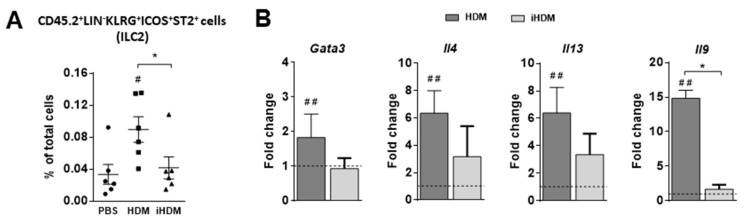
Mice were administered intragastrically PBS or proteolytically active or inactive house dust mite (respectively, HDM and iHDM) for six consecutive days. (**A**) Group 2 innate lymphoid cells (ILC2s, defined as KLRG1^+^ICOS^+^ST2^+^ cells within the CD45.2^+^Lineage^−^ (CD3^−^CD45R^−^CD11b^−^TER-119^−^Ly-G6^−^CD19^−^ cells)) in the lamina propria. (**B**) Relative gene expression of *Gata3*, *Il4*, *Il13*, and *Il9* determined in the jejunum. Gene expression was normalized to the reference gene *Actb* and the mouse group administered PBS, used as calibrator, is represented as a discontinuous line in the figure. Data are expressed as means ± SEM (*n* = 6). Pounds and asterisks indicate, respectively, statistically significant differences with respect to mice administered PBS or between both experimental groups. * and # *p* < 0.05, ## *p* < 0.01.

As IL-33, IL-25, and TSLP drive the expansion of ILC2s at mucosal surfaces [15], we analyzed the proportion of these cells in the lamina propria of mice (Figure 2A). Indeed, ILC2s were enhanced in mice exposed to HDM, while no significant differences were observed in mice fed iHDM as compared with mice administered PBS. ILC2s, that play an important role in mediating oral sensitization to food allergens [26], are governed by the transcription factor GATA-3 and produce Th2 cytokines, such as IL-4, IL-5, IL-13, and IL-9 [27]. Oral administration of HDM increased the expression of *Gata3*, *Il4*, *Il13*, and *Il9* both in the jejunum (Figure 2B) and duodenum of mice, although, in the latter, the level of *Il9* did not reach statistical significance (Supplementary Figure S1). As expected, given that the expression of *Il33*, *Il25*, and *Tslp* and the proportion of ILC2s were not altered, these differences were not observed following the administration of iHDM, except for the fact that *Il13* was upregulated in the duodenum (Figure 2B and Figure S1).

**Figure 3:**
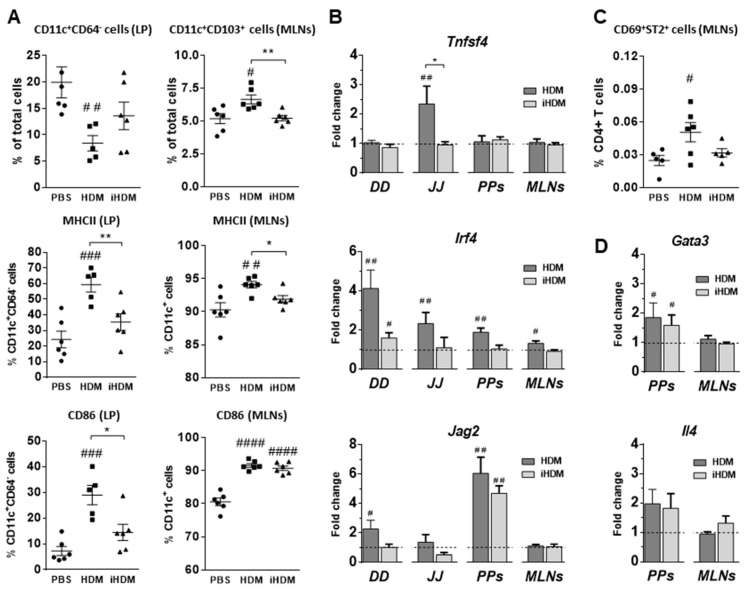
Mice were administered intragastrically PBS or proteolytically active or inactive house dust mite (respectively, HDM and iHDM) for six consecutive days. (**A**) Dendritic cells (DCs) in the lamina propria (LP) (defined by the expression of CD11c^+^ and CD64^−^), LP-derived DCs in the mesenteric lymph nodes (MLNs) (defined by the expression of CD11c^+^ and CD103^+^), and expression of MHCII and CD86 within DCs. (**B**) Relative gene expression of *Tnfsf4*, *Irf4*, and *Jag2* determined in different tissues (duodenum, DD; jejunum, JJ; Peyer’s Patches PPs, and MLNs). (**C**) Activated (CD69^+^) ST2^+^ cells within CD4^+^ T cells in the MLNs. (**D**) Relative gene expression of *Gata3* and *Il4* determined in the PPs and MLNs. Gene expression was normalized to the reference gene *Actb* and the mouse group administered PBS, used as calibrator, is represented as a discontinuous line in the figure. Data are expressed as means ± SEM (*n* = 6). Pounds and asterisks indicate, respectively, statistically significant differences with respect to mice administered PBS or between both experimental groups. * and # *p* < 0.05, ** and ## *p* < 0.01; ### *p* < 0.001; #### *p* < 0.0001.

We then assessed the levels of DCs and the expression of DC genes involved in Th2 differentiation, such as *Tnfsf4*, *Irf4*, and *Jag2*, in nonlymphoid and lymphoid intestinal tissues. The percentage of DCs (cells bearing the surface marker CD11c^+^ but lacking CD64 expression which characterizes macrophages) [28] was reduced in the small intestinal lamina propria of mice given HDM as compared with those fed PBS (Figure 3A). This observation suggested the migration of DCs to lymphoid intestinal tissues, which was confirmed by the increased proportion of CD11c^+^ expressing the integrin *α*-chain CD103, a marker of lamina propria-derived DCs [29], found in the MLNs (Figure 3A). Furthermore, HDM administration induced DC maturation in the intestine, as judged by the upregulation of MHCII and CD86 (Figure 3A). 

A higher proportion of DCs expressing MHCII and CD86 was also observed in the MLNs from mice fed the active extract as compared with control mice although, interestingly, expression of CD86 was not distinctively associated with its proteolytic activity (Figure 3A). Expression of *Tnfsf4*, which encodes OX40L, a costimulatory signal for optimal primary and memory Th2 responses in vivo [30], was increased in the jejunum of mice fed HDM but not in those fed iHDM, although it remained unchanged in the PPs and MLNs (Figure 3B). Moreover, *Irf4*, which encodes the interferon regulatory factor 4 (IRF4), involved in the development and functional specialization of DCs and in the promotion of Th2 differentiation [31], was upregulated in the duodenum, jejunum, PPs, and MLNs of mice exposed to HDM (Figure 3B). The expression of *Jag2*, coding for the Notch ligand Jagged 2, another Th2-skewing factor [32] was also increased in the duodenum of mice fed HDM (Figure 3B). Noteworthy, an enhanced expression of *Jag2* was observed in the PPs of mice fed both HDM and iHDM (Figure 3B). These results suggest that, despite the deleterious effects of HDM on markers of epithelial barrier integrity, promoting the intestinal expression of genes encoding Th2-biasing cytokines and eventually causing ILC2 expansion, upregulation of Th2 cytokines, and CD103^+^ DC migration, all linked to its proteolytic activity, in lymphoid tissues, iHDM, its proteolytically inactivated counterpart, also exerted an influence on the expression of surface DC molecules involved in interactions with T cells and in Th2 cell differentiation, such as CD86 and Jagged 2.

An increased percentage of activated (CD69^+^) CD4^+^T cells bearing the IL-33 receptor ST2, typical of many cell types involved in type 2 immunity and preferentially expressed in Th2 cells [33], was observed in the MLNs of mice fed HDM (Figure 3C). Expression of *Gata3* was significantly enhanced in the PPs of mice administered HDM, although no differences were found with respect to those fed iHDM, nor in the MLNs in comparison with control mice. Similarly, the expression of *Il4* was unchanged both in the PPs and MLNs (Figure 3D).

### 3.3. House Dust Mite causes Dendritic Cell Activation In Vitro Irrespectively of Its Proteolytic Activity

**Figure 4:**
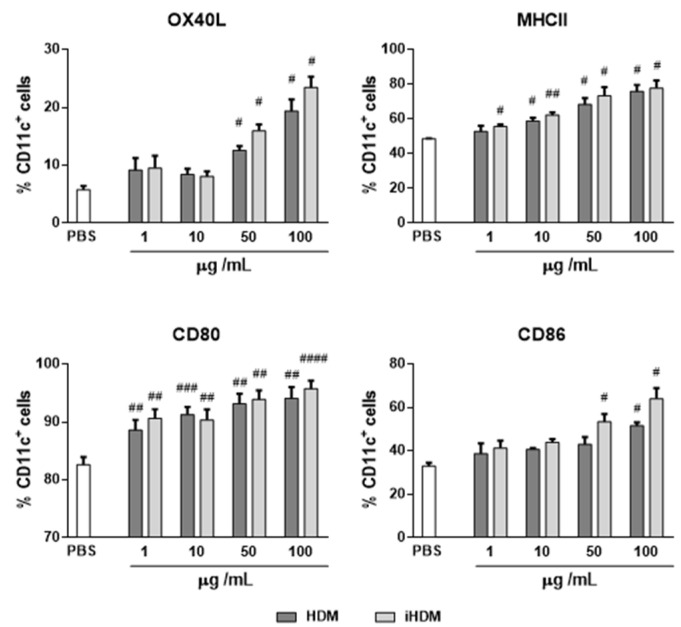
Expression of antigen presentation and costimulatory molecules by bone marrow-derived dendritic cells stimulated with PBS or proteolytically active or inactive house dust mite (respectively, HDM and iHDM) at 1–100 µg/mL for 24 h. Data are expressed as means ± SEM (*n* = 3). Pounds indicate statistically significant differences with respect to mice administered PBS. # *p* < 0.05, ## *p* < 0.01, ### *p* < 0.001, #### *p* < 0.0001.

We next tried to corroborate the response of DCs to HDM and iHDM by conducting in vitro experiments using BM-DCs. Initially, to confirm that the HDM extract contained a low level of exogenous endotoxin, we verified that the amount of LPS present in solution at the concentrations used in this study had no effect on THP1-XBlue cells. Both, HDM and iHDM showed levels of LPS under 0.25 EU/mL (not shown). Flow cytometry analyses revealed that incubation of BM-DCs with both HDM and iHDM, at different concentrations, similarly increased the expression of costimulatory molecules and maturation markers such as OX40L, MHCII, CD80, and CD86 (Figure 4).

**Figure 5:**
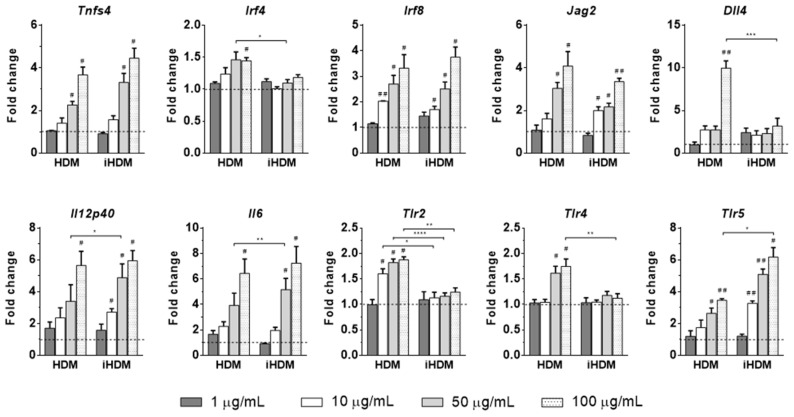
Relative gene expression of *Tnfsf4*, *Irf4*, *Irf8*, *Jag2*, *Dll4*, *Il12p40*, *Il6*, *Tlr2*, *Tlr4*, and *Tlr5* in bone marrow-derived dendritic cells from naïve mice after stimulation with proteolytically active or inactive house dust mite (respectively, HDM and iHDM) at 1–100 µg/mL for 24 h. Gene expression was normalized to the reference gene *Actb* and the mouse group administered PBS, used as calibrator, is represented as a discontinuous line in the figure. Data are expressed as means ± SEM (*n* = 3). Pounds and asterisks indicate, respectively, statistically significant differences with respect to cells cultured in DMEM or between both experimental groups. * and # *p* < 0.05, ** and ## *p* < 0.01, *** *p* < 0.001, **** *p* < 0.0001.

Likewise, expression of *Tnfsf4*, *Jag2, Irf8*, *Il12p40* (the two latter genes encoding the Th1-polarizing factor IRF8 [34] and the Th1 cytokine IL-12), and *Il6* was upregulated in BM-DCs regardless of the proteolytic activity of the extract, although increased expression of *Irf4* and *Dll4* (coding for the Notch ligand Delta 4, which primes naïve T cells for Th1 responses) [34] was only observed in cells stimulated with HDM (Figure 5). This shows that, independently of its proteolytic activity, the extract was able to stimulate the expression of DC genes involved in both Th2 and Th1 responses. Regarding the expression of *Tlr2, Tlr4*, and *Tlr5* (that code, respectively, for the extracellular Toll-like receptors TLR2, TLR4, and TLR5), culture of BM-DCs with HDM, but not with iHDM, enhanced the expression of *Tlr2* and *Tlr4*; while *Tlr5* expression was also upregulated by iHDM, being comparatively higher when the inactivated extract was used for stimulation at its highest concentration (Figure 5).

### 3.4. House Dust Mite Does Not Promote Sensitization to Coadministered Food Allergens

To determine if HDM could provide Th2 adjuvant stimuli and promote sensitization to food allergens, we fed mice with HDM or iHDM together with EW for 6 days, including a final boost administration 2 h after the last dose. Despite the previously described intestinal effects of HDM on the regulation of tight junction proteins and on factors that trigger inflammation and Th2 immunity, associated to its proteolytic activity, no differences were detected when mice were given EW in combination with HDM in the expression in any of the studied genes or in the expansion of ILC2s (Figure S3). Coadministration of EW and HDM seemed to induce migration of DCs from the lamina propria to the MLNs, although the differences did not reach statistical significance. Nevertheless, EW plus HDM and EW plus iHDM upregulated MHCII expression on lamina propria DCs and CD86 expression on MLN DCs, although they did not modulate the expression of the DC Th2-skewing factors *Tnfsf4* or *Irf4* with respect to EW alone. These results thus indicate that the joint administration of EW and HDM largely eliminated the protease-mediated effects of the latter, whereas it did not inhibit the protease-independent, TLR-dependent responses.

**Figure 6:**
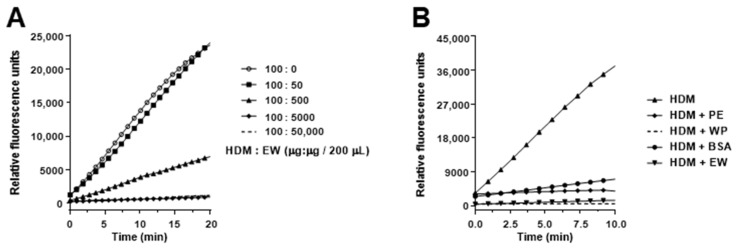
Proteolytic activity (expressed as arbitrary fluorescence units) of house dust mite (HDM). (**A**) Mixed with egg white (EW) in different proportions. (**B**) Mixed with pea protein (PE), whey protein (WP), bovine serum albumin (BSA), or EW in the same proportion as given to the mice (100 µg of HDM plus 50 mg of protein in 200 µL).

As EW contains several protease inhibitors (ovomucoid and ovoinhibitor for serine proteinases, cystatin for cysteine proteinases, and ovostatin for all proteinases) [35], we speculated that they could affect the proteolytic activity of the extract. Incubation of HDM with EW dose-dependently reduced the proteolytic activity of HDM and their combination in the same proportion as that administrated to mice (100 µg of HDM plus 50 mg of EW in 200 µL) completely abolished it (Figure 6A). However, this effect was also observed with other food-derived extracts, such as pea and whey protein, and with BSA, devoid of protease inhibitory activity (Figure 6B), suggesting that, rather than suppression of the proteolytic activity, there was a competition with the fluorogenic peptide substrate used in the assay. In fact, incubation of ovalbumin with HDM at 37 °C overnight led to a small but noticeable proteolytic degradation, as judged from the reduction in the peak corresponding to the protein, detected by RP-HPLC (Figure S4).

## 4. Discussion

IECs and mucus are not only physical barriers protecting from ingested antigens and pathogens, but they can also mount innate immune responses against these agents in the digestive tract [36]. Their disruption, leading to permeation of luminal allergenic or proinflammatory substances, induces activation of the underlying mucosal immune system triggering adaptive responses. Therefore, factors that favor allergen passage through the weakening of the intestinal barrier could facilitate the development of food allergy [14]. Furthermore, it has been postulated that difficulty to appropriately and timely downregulate inflammation, rather than a tendency to mount Th2 responses to antigens, is the cause of deviations from the tolerogenic status [37].

In this work, we showed that orally administered HDM, by virtue of its proteolytic activity, affected the expression of genes that code for tight junction proteins and proinflammatory cytokines (IL-6 and IL-22) in the small intestine, initiating the production of IL-33, IL-25 and TSLP. These function as alarm signals activating ILC2s to produce the Th2 cytokines IL-4, IL-13 and IL-9, with critical roles in type 2 immunity and allergy. Accordingly, and unlike iHDM, proteolytically active HDM also differentially upregulated DC genes coding for Th2 polarizing factors, such as OX40L, IFR4, and Jagged 2. Moreover, HDM induced lamina propria DC maturation and migration to the MLNs, behaving similarly to potent oral Th2 adjuvants such as cholera toxin [34].

Several authors have indicated that the expression of epithelial tight junction molecules, important mucus genes, and barrier function are impaired in the lung of asthmatic patients [38]. Tulic et al. (2016) also showed that HDM could be a potential contributor to intestinal epithelial barrier dysfunction, degrading the tight junctional network and the mucus layer in the colon [11]. In agreement with these authors, we demonstrate that HDM proteases can survive gastrointestinal conditions and remain functional. Our study concentrated on the small intestine, where antigen absorption takes place and the balance between allergy and tolerance is primarily sustained. At the duodenum, but particularly at the jejunum, we observed the upregulation of genes encoding for the pore-forming protein claudin-2, but also for claudin-4, with a sealing function, and for zonula occludens proteins 1 and 2, that provide the intracellular scaffold [39]. This may indicate a compensatory defense response induced by the proteolytic disruption of the tight junction proteins, as shown by other authors who found a transient upregulation of tight junction genes during early stages of acute lung or oral epithelial cell injury [38,40]. In our study, expression of *Muc2* was not modified by HDM administration, even if we detected upregulation of the gene encoding IL-22, which is known to induce expression of mucins as part of its protective role in the gastrointestinal tract [23]. In this respect, it should be noted that Muc2 is much more abundant in the large than in the small intestine where its structure and function are not fully clear [36].

The distinct influence exerted by HDM as compared with iHDM on factors that govern epithelial barrier integrity and intestinal cytokine responses supports a putative causal relationship between intestinal epithelial effects triggered by the active extract and the induction of type 2 immunity. However, iHDM also stimulated DCs, in vivo in the PPs and MLNs as well as in vitro, upregulating T cell costimulatory molecules and genes that drive both Th1 and Th2 responses.

It is known that HDM, independently of its proteolytic activity, also acts as a strong activator of innate immune cells through PRR-dependent mechanisms triggered by contaminating or endogenous PAMPs. This leads to the upregulation of proinflammatory and pro-Th2 cytokines and chemokines that are released, not only to recruit and activate inflammatory cells, but also to induce Th2 differentiation [3]. Indeed, the expression of the genes that code for IL-6, IL-12, Notch ligands from the Jagged and Delta family, and the transcription factors IFR4 and IFR8 is activated downstream a pathway that depends on MyD88, an adaptor protein that is conventionally associated with TLR signaling [32,41,42]. The contribution of activation of innate immunity via TLR4 and TLR2 signaling by microbial compounds comprised in HDM, such as LPS and *β*-glucans, to initiate Th2 polarization and HDM allergy was clearly confirmed. The relative importance of TLR4 or TLR2 activation is tissue-dependent, due to the specific role of LPS or *β*-glucans in inducing TLR4 and TLR2 surface expression and translocation into lipid rafts in the lower and upper airways, respectively [43]. In addition, Der p 2 has structural and functional homology with LPS and *Dermatophagoides farinae* 2 (Der f 2) and presents lipid-binding properties that enable it to transport lipids other than LPS that constitute TLR2 ligands [44]. Therefore, both Der p 2 and Der f 2, can promote airway inflammation in TLR4- and TLR2-dependent manners under conditions of very low levels of LPS exposures [9]. Similarly, stimulation of TLR5 on DCs by bacterial flagellin induces Th2 responses that have been associated with the prevalence of allergic rhinitis, although, similarly to other TLR ligands, high doses can induce tolerogenic responses [45]. Therefore, it can be hypothesized that TLR stimulation of intestinal innate cells, including DCs, may constitute a mechanism through which ingested HDM could induce oral sensitization.

Expression of TLRs was previously shown to be directly upregulated by TLR stimulation [41] and incubation of BM-DCs with the active extract significantly upregulated *Tlr2*, *Tlr4*, and *Tlr5*. The reason why proteolytically active HDM, but not the inactive form, enhanced the expression of *Tlr2* and *Tlr4* remains to be elucidated. TLR4 has been reported to respond, not only to bacterial LPS, but also to an array of other exogenous and endogenous agonists, including the serine protease elastase [46]. On the other hand, concurrent activation of PARs and TLR4 amplifies NF-κB activation, revealing the existence of an interaction between both types of receptors [47]. Interestingly, iHDM significantly enhanced *Tlr5* expression over HDM, which could contribute to reinforce TLR-mediated effects in the absence of proteolytic activity.

In our experimental setting, we did not find evidence for HDM exerting an amplifying effect on type 2-promoting responses induced by a common food allergen such as EW, except for an increased DC activation (as shown by an enhanced expression of antigen presentation -MHCII- and costimulatory molecules -CD86-). Since HDM upregulates tissue damage factors and acts as an activator of innate immune cells, behaving similarly to oral Th2 adjuvants, it cannot be discarded that HDM, either orally ingested or swallowed from the upper airways, could prime the immune system for subsequent sensitization to food allergens. However, the joint administration of low amounts of HDM in relation to food proteins most likely causes proteolytic degradation of the latter, minimizing its gastrointestinal effects.

## Supplementary Materials

The following are available online at https://www.mdpi.com/2304-8158/10/3/561/s1, Table S1: Primer pair sequences for the analyses of gene expression, Figure S1: Relative gene expression of Cldn2, Cldn3, Tjp1, Tjp2, Il22, Il33, Il25, Tslp, Il4, Il13, and Il9 determined in the duodenum of mice administered intragastrically proteolytically active or inactive HDM, Figure S2: Proteolytic activity of HDM at pH 2.0, 3.5 and 7.0, Figure S3: Gene expression, Group 2 innate lymphoid cells, and dendritic cells from mice administered intragastrically with egg white alone or in combination with proteolytically active or inactive HDM, Figure S4: RP-HPLC analyses of ovalbumin, as such, and after incubation with HDM.
