# Supplementary material for: Oral Exposure to House Dust Mite Activates Intestinal Innate Immunity
Source: Foods. 2021 Mar 9;10(3):561. doi: 10.3390/foods10030561 (PMC8000190; doi:10.3390/foods10030561)
Supplement: Supplementary file 1 [file foods-10-00561-s001.pdf]

Supplementary Table 1: *Primer pair sequences for the analyses of gene expression.*

Gene	Primer pairs	Reference	Cycling conditions
Actb	*fw* 5' AGCTGCGTTTTACACCCTTT 3'<br>*rv* 5' AAGCCATGCCAATGTTGTCT 3'	[Cardoso et al., 2009]	*Pre-Incubation*<br>*2 min 50ºC*<br><br>*Incubation*<br>*10 min 95ºC*<br><br>**<u>40 cycles:</u>**<br><br>*Denaturation*<br>*15 s 95ºC*<br><br>*Annealing/Extension*<br><br>45 s 58ºC + 15 s 60ºC
Cldn2	*fw* 5' GTCATCGCCCATCAGAAGAT 3'<br>*rv* 5' ACTGTTGGACAGGGAACCAG 3'	[Volvolynets et al., 2016]	
Cldn3	*FW 5' CCACTACCAGCAGTCGATGA 3'*<br>*RV 5' CAGCCTGTCTGTCCTCTTCC 3'*	[Corridoni et al., 2012]	
Tjp1	*FW 5' TACCTCTTGAGCCTTGAACTT 3'*<br>*RV 5' ACAGAAATCGTGCTGATGTGC 3'*	[Lozano-Ojalvo et al., 2019]	
Il22	*fw* 5' CATGCAGGAGGTGGTACCTT 3'<br>*rv* 5' CAGACGCAAGCATTTCTCAG 3'	[Lozano-Ojalvo et al., 2019]	
Il6	*fw* 5' TTCCATCCAGTTGCCTTCTTG 3'<br>*rv* 5' GGGAGTGGTATCCTCTGTGAAGTC 3'	[Tordesillas et al., 2014]	
Il4	*fw* 5' CCTCACAGCAACGAAGAACA 3'<br>*rv* 5' ATCGAAAAGCCCGAAAGAGT 3'	[Yang et al., 2009]	
Il13	*fw* 5' CATGGCCTCTGTAACCGCAA 3'<br>*rv* 5' CCTCATTAGAAGGGGCCGTG 3'	[Pérez-Rodriguez et al., 2020]	
Tlr2	*fw 5' TCTGCGACCTAGAAGTGGAA 3'*<br>*rv 5' TGAAGCCCCTACACTCAGAA 3'*	This work	
Tlr4	*fw 5' AATGCCCTATTGGATGGAAA 3'*<br>*rv 5' AGGCCCCAGAGTTTTGTTCT 3'*	[Benedé et al., 2018]	
Tlr5	*fw 5' GGTGTGATCTTCATGGCCAGCCC 3'*<br>*rv 5'CGTCGCTTAAGGAATTCAGTTCCCGG3'*	[Mathur et al., 2012]	
Actb	*fw* 5' AGCTGCGTTTTACACCCTTT 3'<br>*rv* 5' AAGCCATGCCAATGTTGTCT 3'	[Cardoso et al., 2009]	*Pre-Incubation*<br>*2 min 50ºC*<br><br>*Incubation*<br>*10 min 95ºC*<br><br>**<u>40 cycles</u>:**<br><br>*Denaturation*<br>*15 s 95ºC*<br><br>*Annealing/Extension*<br>*60 s 60ºC*
Il33	*fw* 5' ATTTCCCCGGCAAAGTTCAG 3'<br>*rv* 5' AACGGAGTCTCATGCAGTAGA 3'	[Li et al., 2013]	
Il25	*fw* 5' ACAGGGACTTGAATCGGGTC 3'<br>*rv* 5' TGGTAAAGTGGGACGGAGTTG 3'	[Li et al., 2013]	
Tslp	*fw* 5' AGGCTACCCTGAAACTGAGA 3'<br>*rv* 5' GGAGATTGCATGAAGGAATAC 3'	[Negishi et al., 2012]	
Gata3	*fw* 5' CCTTAAAACTCTTGGCGTCC 3'<br>*rv* 5' AGACACATGTCATCCCTGAG 3'	[Zhang et al., 2013]	
Il9	*fw* 5' GTCCGTCCTTTTCCTGCGAA 3'<br>*rv* 5' TCTGTCTTCATGGTCGGCTT 3'	This work	
IL12p 40	*fw 5' AGGTGCGTTCCTCGTAGAGA 3'*<br>*rv 5' AAAGCCAACCAAGCAGAAGA 3'*	[Blazquez and Berin, 2016]	
Jag2	*fw* 5' GGCAAAGAATGCAAAGAAGC 3'<br>*rv* 5' GCTCAGCATTGATGCAGGTA 3'	[Blazquez and Berin, 2016]	
Irf4	*fw* 5' TCCTCGTCCCTTGCTGAAAC 3'<br>*rv* 5' GGGCTTTGGGGCTTCTAGTT 3'	[Pérez-Rodriguez et al., 2020]	
Actb	*fw* 5' AGCTGCGTTTTACACCCTTT 3'<br>*rv* 5' AAGCCATGCCAATGTTGTCT 3'	[Cardoso et al., 2009]	*Pre-Incubation*<br>*2 min 50ºC*<br><br>*Incubation*<br>*10 min 95ºC*<br><br>**<u>40 cycles:</u>**<br><br>*Denaturation*<br>*15 s 95ºC*<br><br>*Annealing/Extension*<br>*30 s 56ºC + 30 s 58ºC*
Tjp2	*FW 5' TGGGACCGTCGCTTTCTG 3'*<br>*RV 5' CTGTGGCGGGGAGGTTTGA 3'*	This work	
Tnfsf4	*fw* 5' GGGATGCTTCTGTGCTTCATCT 3'<br>*rv* 5' TTTGGATTGGAGGGTCCTTTG 3'	[Mehta et al., 2016]	

fw, forward; rv, reverse

Cardoso, C.R., Provinciatto, P.R., Godoi, D.F., Ferreira, B.R., Teixeira, G., Rossi, M.A. (2010). IL-4 regulates susceptibility to intestinal inflammation in murine food allergy. *American Journal of Physiology - Gastrointestinal and Liver Physiology*, 296: 593-600.

Volynets, V., Rings, A., Bárdos, G., Ostaff, M.J., Wehkamp, J., Bischoffa, S.C. (2016). Intestinal barrier analysis by assessment of mucins, tight junctions, and α-defensins in healthy C57BL/6J and BALB/cJ mice. *Tissue Barriers.* 4: 15-30.

Corridoni, D., Pastorelli, L., Mattioli, B., Locovei, S., Ishikawa, D., Arseneau, K.O., Chieppa, M., Cominelli, F., Pizarro, T.T. (2012). Probiotic Bacteria Regulate Intestinal Epithelial Permeability in Experimental Ileitis by a TNF-Dependent Mechanism**.** *Plos One.* 7(7): e42067.

Lozano-Ojalvo D, Martínez-Blanco M, Pérez-Rodríguez L, Molina E, Peláez C, Requena T, López-Fandiño R. 2019. Egg white peptide-based immunotherapy enhances vitamin A metabolism and induces RORγt+ regulatory T cells. Journal of Functional Foods. 52, 204-211.

Tordesillas, L., Goswami, R., Benedé, S., Grishina, G., Dunkin, D., Järvinen, K.M., Maleki, S.J., Sampson, H.A., Berin, M.C. (2014). Skin exposure promotes a Th2-dependent sensitization to peanut allergens. *The Journal of Clinical Investigation,* 124: 4965-4975.

Li, J., Wang, Y., Tang, L., Villiers, W.J., Cohen, D., Woodward, J., Finkelman, F.D., Eckhardt, E.R. (2013). Dietary medium-chain triglycerides promote oral allergic sensitization and orally induced anaphylaxis to peanut protein in mice. *Journal of Allergy and Clinical Immunology*, 131: 442-50.

Negishi, H., Miki, S., Sarashina, H., Taguchi-Atarashi, N., Nakajima, A., Matsuki, K., Endo, N., Yanai, H., Nishio, J., Honda, K., Taniguchi, T. (2012). Essential contribution of IRF3 to intestinal homeostasis and microbiota-mediated Tslp gene induction. *Proceedings of the National Academy of Sciences*, 109: 21016-21021.

Zhang, C., Gui, L., Xu, Y., Wu, T., and Liu, D. (2013). Preventive effects of andrographolide on the development of diabetes in autoimmune diabetic NOD mice by inducing immune tolerance. *Internacional Immunopharmacology*, 16: 451-456.

Yang, M., Yang, C., Nau, F., Pasco, M., Juneja, L.R., Okubo, T., Mine, Y. (2009). Immunomodulatory effects of egg white enzymatic hydrolysates containing immunodominant epitopes in a Balb/c mouse model of egg allergy. *Journal of Agricultural and Food Chemistry,* 57: 2241-2248.

Pérez-Rodríguez, L., Martínez-Blanco, M., Lozano-Ojalvo, D. et al. Egg yolk augments type 2 immunity by activating innate cells. Eur J Nutr (2020).

Blazquez, A.B., Berin, C. (2016). Gastrointestinal Dendritic Cells Promote Th2 Skewing via OX40L. *The Journal of Immunology*, 180: 4441-4450.

Mehta, A.K., Duan, W., Doerner, A.M., Traves, S.L., Broide, D.H., Proud, D., Zuraw, B.L., Croft, M. (2016). Rhinovirus infection interferes with induction of tolerance to aeroantigens through OX40 ligand, thymic stromal lymphopoietin, and IL-33. *Allergy and Clinical Immunology*, 137: 278-288.

Benedé, S., Cody,E., Agashe, C., Berin, C. (2018). Immune Characterization of Bone Marrow-Derived Models of Mucosal and Connective Tissue Mast Cells. *Allergy Asthma Immunology Research.* 10: 268–277.

Mathur, R., Oh, H., Zhang, D., Park, S.G., Seo, J., Koblansky, A., Hayden, M.S., Ghosh, S. (2012). A Mouse Model of Salmonella Typhi Infection. *Cell.* 151: 590-602.

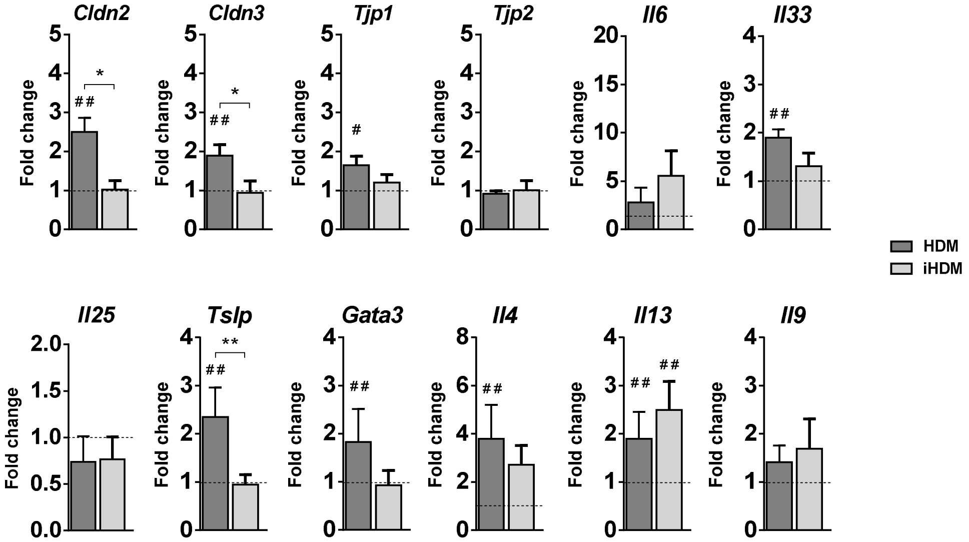

Supplementary Figure 1. Relative gene expression of *Cldn2*, *Cldn3*, *Tjp1*, *Tjp2*, *Il22*, *Il33*, *Il25*, *Tslp*, *Il4*, *Il13*, and *Il9* determined in the duodenum of mice administered intragastrically proteolytically active or inactive house dust mite (respectively HDM and iHDM) for 6 consecutive days. Gene expression was normalized to the reference gene *Actb*. Data are expressed as means ±SEM (n=6). Dashed lines indicate the reference value for mice administered PBS. Pounds and asterisks indicate, respectively, statistically significant differences with respect to mice administered PBS or between both experimental groups. * and # p<0.05, ** and ## p<0.01.

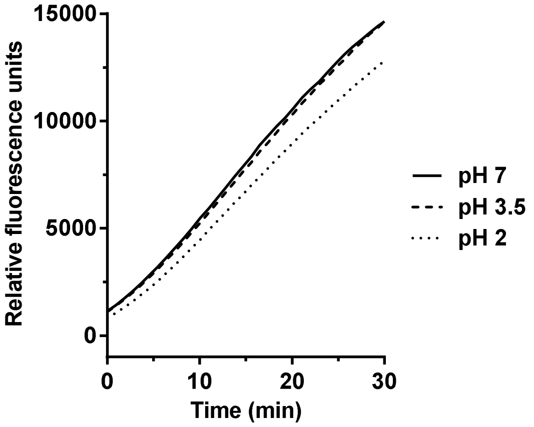

Supplementary Figure 2. Proteolytic activity (expressed as arbitrary fluorescence units) of house dust mite (HDM) at pH 2.0, 3.5 and 7.0.

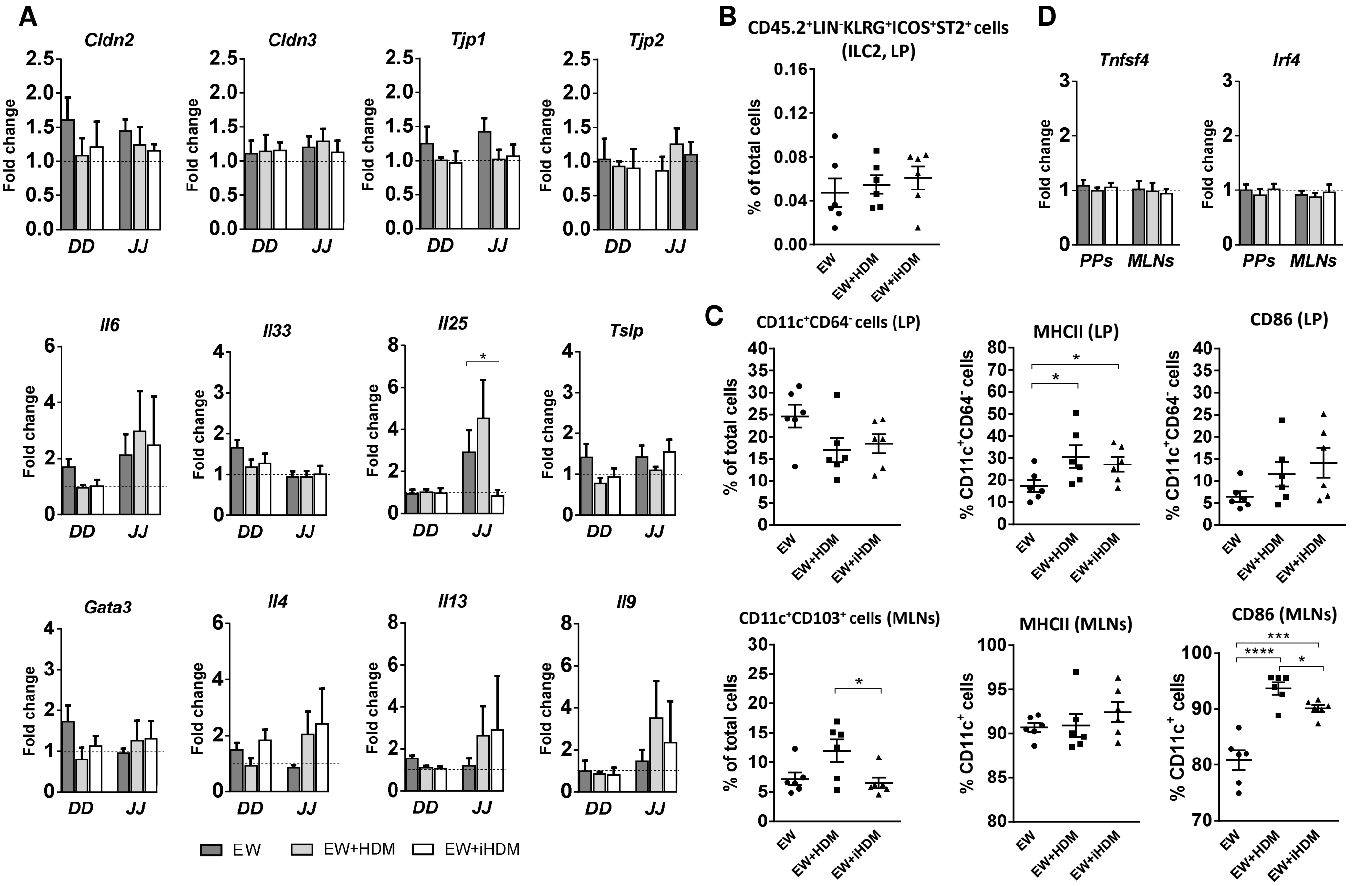

Supplementary Figure 3. Mice were administered intragastrically egg white (EW) alone or in combination with proteolytically active or inactive house dust mite (respectively EW+HDM and EW+iHDM) for 6 consecutive days. **A** Relative gene expression of *Cldn2*, *Cldn3*, *Tjp1*, *Tjp2*, *Il6*, *Il33*, *Il25*, *Tslp*, *Gata3*, *Il4*, *Il13*, and *Il9* determined in the duodenum (DD) and jejunum (JJ). **B** Group 2 innate lymphoid cells (ILC2s, defined as $KLRG1^{+}ICOS^{+}ST2^{+}$ cells within the $CD45.2^{+}$ $Lineage^{-}$ [$CD3^{-}CD45R^{-}CD11b^{-}$ $TER\text{-}119^{-}Ly\text{-}G6^{-}CD19^{-}$ cells]) in the lamina propria (LP). **C** Dendritic cells (DCs) in the LP (defined by the expression of $CD11c^{+}$ and $CD64^{-}$), LP-derived DCs in the mesenteric lymph nodes (MLNs) (defined by the expression of $CD11c^{+}$ and $CD103^{+}$), and expression of MHCII and CD86 within DCs. **D** Relative gene expression of *Tnfs4* and *Irf4* determined in Peyer's Patches (PPs) and MLNs). Gene expression was normalized to the reference gene *Actb*. Data are expressed as means ± SEM (n=6). Dashed lines indicate the reference value for mice administered PBS. Asterisks indicate statistically significant differences among groups. * $p<0.05$, ***$p<0.001$, ****$p<0.0001$.

Supplementary Figure 4

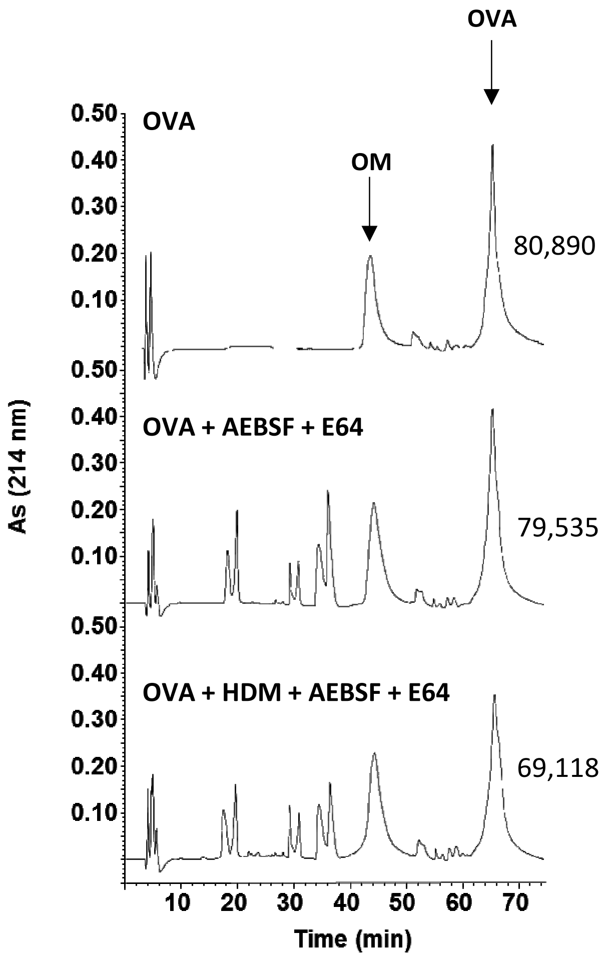

Supplementary Figure 4. RP-HPLC analyses of ovalbumin (OVA), as such, and after incubation with house dust mite (HDM) for 24 h at 37 ºC, followed by the addition of the serine protease inhibitor AEBSF and the cysteine protease inhibitor E-64. The effect of the sole addition of inhibitors is also shown for comparison. Ovomucoid impurity (OM). Numbers indicate integration values of OVA peaks (mV*sec).
